# High-Definition Transcranial Direct Current Stimulation (HD-tDCS) Therapy in Amyotrophic Lateral Sclerosis: Study Protocol for a Multicenter Randomized Controlled Clinical Trial

**DOI:** 10.3390/jcm14196701

**Published:** 2025-09-23

**Authors:** Edna Karla Ferreira Laurentino, Vinicius Zacarias Maldaner da Silva, Wesley Ribeiro Costa Meneses, Lariza Maria da Costa, Matias Otto-Yañez, Roberto Vera-Uribe, Rodrigo Torres-Castro, Bruna Ribeiro Carneiro de Sousa, Rodrigo Pegado de Abreu Freitas, Sergio Ricardo Menezes Mateus, Ingrid Faber de Vasconcellos, Hamilton Cirne Fernandes Franco, Danilo Alves Pinto Nagem, Ricardo Alexsandro de Medeiros Valentim, Mário Emílio Dourado Júnior, Ana Raquel Rodrigues Lindquist, Suellen Mary Marinho dos Santos Andrade, Jéssica D. Medeiros Fonseca, Vanessa Regiane Resqueti, Guilherme de Freitas Fregonezi

**Affiliations:** 1 PneumoCardioVascular Lab/HUOL, Departamento de Fisioterapia, Hospital Universitário Onofre Lopes, Universidade Federal do Rio Grande do Norte, Natal 59010-090, Rio Grande do Norte, Brazil; edna.karla.117@ufrn.edu.br (E.K.F.L.); wesley.meneses.063@ufrn.edu.br (W.R.C.M.); lariza.costa.121@ufrn.edu.br (L.M.d.C.); jessy_nielle@hotmail.com (J.D.M.F.); vanessa.resqueti@ufrn.br (V.R.R.); 2 Laboratório de Inovação Tecnológica em Reabilitação, Departamento de Fisioterapia, Universidade Federal do Rio Grande do Norte, Natal 59012-300, Rio Grande do Norte, Brazil; 3 Ceilândia Sul—Campus Universitário, Universidade de Brasília, Centro Metropolitano, Ceilândia 72220-275, Distrito Federal, Brazil; viniciusmaldaner@gmail.com (V.Z.M.d.S.); srmmateus@gmail.com (S.R.M.M.); 4 Escuela de Kinesiologia, Universidad Autonoma de Chile, Santiago 7501012, Chile; matiasotto.kine@gmail.com; 5 Departamento de Kinesiología, Facultad de Medicina, Universidad de Chile, Santiago 8380453, Chile; robertovera@uchile.cl (R.V.-U.); klgorodrigotorres@gmail.com (R.T.-C.); 6 Departamento de Fisioterapia, Universidade Federal do Rio Grande do Norte, Natal 59078-900, Rio Grande do Norte, Brazil; bruna.sousa.106@ufrn.edu.br (B.R.C.d.S.); raquel.lindquist@ufrn.br (A.R.R.L.); 7 Programa de Pós-Graduação em Ciências da Reabilitação, Universidade Federal do Rio Grande do Norte, Natal 59078-900, Rio Grande do Norte, Brazil; rodrigopegado@gmail.com; 8 Hospital Universitário de Brasília, Universidade de Brasília, Brasília 70840901, Distrito Federal, Brazil; ingridfaberneuro@gmail.com; 9 Departamento de Neurologia, Centro de Referência Terciária de Doenças Neuromusculares, Hospital de Apoio de Brasília, Brasília 70684-831, Distrito Federal, Brazil; cirne81@yahoo.com.br; 10 Laboratório de Inovação Tecnológica em Saúde (LAIS), Universidade Federal do Rio Grande do Norte, Natal 59012-300, Rio Grande do Norte, Brazil; danilo.nagem@lais.huol.ufrn.br (D.A.P.N.); ricardo.valentim@lais.huol.ufrn.br (R.A.d.M.V.); 11 Divisão de Neurologia, Hospital Universitário Onofre Lopes, Universidade Federal do Rio Grande do Norte, Natal 59012300, Rio Grande do Norte, Brazil; medourado03@gmail.com; 12 Laboratório de Envelhecimento e Neurociências, Universidade Federal da Paraíba, João Pessoa 59051-900, Paraíba, Brazil; suellenandrade@gmail.com

**Keywords:** neuromuscular diseases, transcranial electrical stimulation, home-based treatment

## Abstract

**Background/Objectives:** Amyotrophic Lateral Sclerosis (ALS) is a progressive and fatal neurodegenerative disease characterized by motor neuron loss, muscle weakness, and respiratory dysfunction, often culminating in ventilatory failure. Evidence suggests that High-Definition Transcranial Direct Current Stimulation (HD-tDCS) may modulate motor cortical excitability and potentially influence motor and respiratory function in ALS. This study aims to evaluate the effects of home-based HD-tDCS applied over the primary diaphragmatic motor cortex on respiratory parameters and disease progression in individuals with ALS. **Methods:** This is a multicenter, randomized, controlled clinical trial. Eligible participants (aged 18–80, both sexes, diagnosed with ALS) will be randomized into an active HD-tDCS group (gTDCS) or a sham group (gSham). The intervention consists of 30 min daily HD-tDCS sessions (3 mA) applied for two weeks (5 days/week), using a 4 × 1 ring configuration targeting the diaphragmatic motor cortex. Sham stimulation includes an identical setup but only delivers ramp currents (30 s) with a minimal ongoing current (0.1 mA). **Results:** Pre-, intra-, and post-intervention evaluations will include measures of cortical excitability, cerebral and tissue perfusion, surface electromyography, respiratory and pulmonary function, fatigue, sleep quality, pain, motor performance, dyspnea, quality of life, and adverse effects. All procedures will be conducted at participants’ homes with appropriate safety monitoring. **Conclusions:** This study will investigate the effects of HD-tDCS on respiratory and motor function in ALS and explore the feasibility of a home-based neuromodulation intervention. The outcomes may provide insight into non-pharmacological strategies for respiratory management in ALS.

## 1. Introduction

Amyotrophic Lateral Sclerosis (ALS) is an acquired, degenerative, and disabling nervous system disease characterized by the loss of motor neurons in the cortex, brainstem, and spinal cord [1]. People with ALS experience muscle weakness that progresses to loss of mobility and functionality, swallowing difficulties, and respiratory muscle dysfunction, and frequently results in death due to ventilatory failure [2]. As such, ALS is one of the most disabling neuromuscular diseases, with rapid and essentially fatal progression. The most common global symptoms faced by people with ALS include sialorrhea, spasticity, muscle atrophy, balance deficits, cognitive impairment, ventilatory insufficiency, increased bronchial secretions, muscle cramps, insomnia, fatigue, and difficulties with communication, swallowing, and nutrition [3,4].

Fundamental aspects of disease progression relate to motor and respiratory function. Muscle weakness progresses from the early stages of the disease, being asymmetrical and variable between the upper and lower limbs, with low resistance to effort and consequently reduced functional performance, which negatively impacts quality of life [5]. Progressive loss of respiratory muscle strength is one of the main challenges for ALS patients. Although this loss plays a significant role in the natural history of the disease, it varies, occurring more rapidly in some individuals and more slowly in others [6].

The death of respiratory motor neurons in ALS leads to denervation and partial reinnervation of respiratory muscle fibers by adjacent motor axons. This collateral reinnervation gives rise to larger, more complex motor units that may partially preserve global respiratory function but compromise more complex motor activities, such as the coordination between breathing and swallowing and the control of sialorrhea [7].

In addition to peripheral failure, respiratory dysfunction in ALS is also manifested by a compensatory increase in the neural drive to breathe, as the central nervous system attempts to maintain ventilation. This neural overload occurs especially during sleep, when the automatic mechanisms of respiratory control are most demanded. Hypoventilation initially develops during the night, associated with sleep-disordered breathing such as sleep apnea and sleep fragmentation, and later extends into the daytime period. This imbalance is one of the main determinants of the need for ventilatory support and directly impacts patients’ quality of life and prognosis. Nocturnal hypoventilation is an early manifestation in ALS patients. Studies have shown that these patients present significantly worse spirometric values and blood gas parameters compared to those without nocturnal hypoventilation, suggesting that the loss of central ventilatory drive contributes to hypopnea and desaturation during sleep [8,9].

As the disease progresses, the continuous loss of respiratory motor neurons leads to muscle weakness and atrophy, resulting in progressive hypoventilation and the development of chronic respiratory failure, a factor directly related to reduced survival in these patients [7]. In patients with chronic obstructive pulmonary disease (COPD), ref. [10] demonstrated basal hyperexcitability in brain regions involved in compensating for respiratory loads, such as the motor and premotor cortices. Nguyen et al. [11] corroborated these findings, highlighting the association between cortical activity related to breathing and the increased perception of respiratory effort during ventilatory loads.

Cortical centers can directly influence spinal motoneurons, allowing indirect cortical control of respiratory muscle activity through the brainstem respiratory center [12,13,14,15,16]. The study by Azabou et al. [15] reported that transcranial direct current stimulation (tDCS) applied to the primary motor cortex was able to modulate the excitability of respiratory neural pathways, showing that anodal or cathodal stimulation in healthy individuals resulted in a reduction in the excitability of diaphragmatic corticospinal pathways. These findings support the potential for neuromodulatory interventions targeting central respiratory control.

In terms of survival, the lack of curative treatments or those that significantly alter the clinical course and progression of ALS has led to the search for new treatment alternatives, proposed in recent years. Over the last two decades, new therapeutic hypotheses have emerged, based on case studies on the application of transcranial direct current stimulation (tDCS). Specifically, different forms of modulation based on non-invasive brain stimulation techniques have been tested and evaluated as non-pharmacological methods for treating various neurological conditions.

tDCS is a brain modulation technique with a strong physiological basis and significant practical acceptance in the treatment of various neurological and musculoskeletal disorders. Studies point to interesting possibilities for using tDCS in treating motor and sensory dysfunctions and learning disorders, making it a potential adjunct to conventional treatments [12,13]. Stimulation can last minutes or even hours after the end of the session. Thus, it emerges as a potential tool for treating motor neuron diseases and degenerative neurological syndromes. Given its ease of application, minimal adverse effects, and relatively low cost, tDCS is clearly a feasible option for routine use in public and supplementary healthcare systems [17,18].

Although widely used, conventional tDCS has evolved into a more advanced form known as high-definition tDCS (HD-tDCS) [19]. This modality features smaller electrodes and a configuration involving a central electrode, with either anodic or cathodic current, surrounded by two or more electrodes with opposite polarity [20,21,22,23]. This configuration helps confine neuromodulation effects to the radius of the four electrodes and increases the focal effect, producing better results in the different areas of the stimulated motor cortex, including the diaphragmatic cortex, to investigate, for instance, changes in muscle strength and perceived exertion [23].

HD-tDCS has been widely investigated as a safe and non-invasive neuromodulation tool [21]. Unlike other forms of electrical stimulation, HD-tDCS does not directly generate action potentials but modulates cortical excitability, making the stimulated region more susceptible to responding to subsequent stimuli [24]. This modulation occurs in a complex manner, depending on the function and basal state of the local neural network, which involves interactions between excitatory and inhibitory neurons [25]. Evidence suggests that anodal tDCS, unlike cathodal tDCS, can induce beneficial functional effects without accelerating the progression of Amyotrophic Lateral Sclerosis (ALS) [26,27]. More recent studies indicate that anodal current applied to the motor cortex, combined with cathodal current to the spinal cord (conventional tDCS), may reduce disease progression [28,29]. Additionally, isolated cathodal tDCS has not demonstrated measurable effects on muscle strength or disease progression [30,31,32].

The study by Benussi et al. [29], using computational modeling of current flow and multichannel tDCS application—consisting of anodal stimulation of the right and left primary motor cortex with the cathodal electrode at the spinal region (C6)—aimed to achieve a synergistic effect from stimulating both structures. In a sample of thirty patients, significant improvements/stabilizations were observed in clinical scores of peripheral muscle strength, quality of life, and caregiver burden after 2 weeks of treatment. The protocol involved applying tDCS for 20 min/day at an intensity of 2 mA. Interestingly, these effects persisted for 6 months after the treatment ended, with clinical parameters and neurophysiological assessment through Transcranial Magnetic Stimulation (TMS) monitoring progress. Additionally, this study reported slower disease progression, expressed by a decline in the Revised Amyotrophic Lateral Sclerosis Functional Rating Scale (ALSFRS-R) score of less than 2 points over 6 months, characterizing the real tDCS and sham/control groups.

While there are studies suggesting neuromodulation effects through tDCS intervention in the ALS population, the effects of this therapy on the respiratory system remain unclear, creating a gap in the literature. Thus, the hypothesis of the present research is that HD-tDCS may stimulate motor areas related to respiratory muscles and produce significant effects on pulmonary function and stabilization of disease progression in individuals with ALS, with an initial focus on neurophysiological and respiratory outcomes.

### 1.1. Objectives

#### 1.1.1. Primary Objective

To evaluate the effects of HD-tDCS on respiratory function and neurophysiological responses associated with stimulation of the diaphragmatic primary motor cortex in individuals with ALS.

#### 1.1.2. Secondary Objectives

▪ To assess cortical excitability by measuring patterns of facilitation, inhibition, active and resting motor thresholds, central motor conduction time, cortical silent period, and Motor Evoked Potentials (MEPs) in individuals with ALS;
▪ To evaluate cortical tissue perfusion in the region of the diaphragmatic primary motor cortex in individuals with ALS through specific respiratory tests before and after the HD-tDCS intervention protocol;
▪ To assess respiratory muscle electrical activity in individuals with ALS before and after the HD-tDCS intervention protocol;
▪ To observe the effects of HD-tDCS on aspects such as respiratory muscle strength, nasal inspiratory and expiratory pressures, and peak cough flow through specific respiratory tests in individuals with ALS;
▪ To observe the effects of HD-tDCS on clinical and functional aspects using the Amyotrophic Lateral Sclerosis Functional Rating Scale—Revised (ALSFRS-R);
▪ To assess motor control and muscle performance using a protocol for evaluating peripheral muscle electrical activity;
▪ To assess fatigue, dyspnea, pain, sleep, quality of life, and cognition before and after the HD-tDCS intervention protocol;
▪ To identify adverse effects of HD-tDCS on the diaphragmatic motor cortex in individuals with ALS.

## 2. Methods

### 2.1. Study Type and Sample

**Figure 1:**
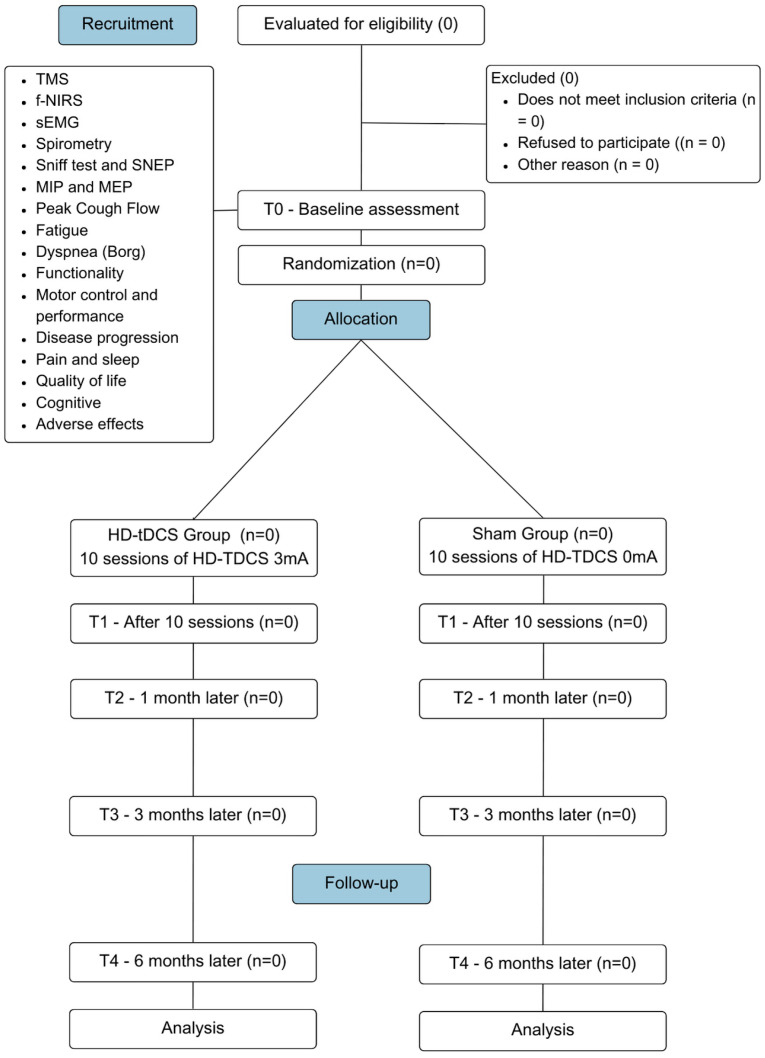
CONSORT flow diagram flowchart illustrating the CONSORT for the study design. This diagram outlines the participant flow through the phases of the multicenter, randomized, controlled, triple-blind clinical trial.

This is a multicenter, parallel, controlled, randomized, triple-blind clinical trial following the criteria set by the Consolidated Standards of Reporting Trials (CONSORT) [33] and Standard Protocol Items Recommendations for Interventional Trials (SPIRIT) (Figure 1) [34]. The study will be conducted between 2025 and 2027 at three leading research centers. The purpose is to analyze the effects of HD-tDCS on the primary diaphragmatic motor cortex in subjects with a clinical diagnosis of ALS.

The study population will consist of patients clinically diagnosed with ALS by a neurologist based on diagnostic tests (clinical aspects, electrodiagnostic, genetic, and neuroimaging) [35] and the criteria established by the El Escorial World Federation of Neurology [36]. The participants will include individuals of both sexes who are being monitored in the outpatient clinics of the study centers.

### 2.2. Sample Recruitment, Randomization, and Blinding

Participants will be recruited by direct invitation to the Neuromuscular Disease Clinic of the University Hospital, where they have already been diagnosed and receive follow-up care from a multidisciplinary team. Only those who meet the eligibility criteria will be included in the study. After being invited to participate and given a clear explanation of all procedures involved in the research, including the possibility of being assigned to the control or experimental group, each individual will have one week to discuss the decision with their family. Following this period and after signing written consent, participants will be taken to the lab for baseline evaluation. Patients will receive interventions at home and will be evaluated at outpatient units and research labs. Being informed of the benefits of conducting tDCS therapy at home may enhance patient adherence to treatment by reducing transportation costs and associated risks.

The sample will consist of individuals with ALS who have signed the Informed Consent Form (ICF) (Supplementary Materials S1) authorizing their participation in the research and meet the study’s inclusion criteria. Participants will be randomized by an external researcher (using sealedenvelope.com) into two groups: the HD-tDCS group (gTDCS) and the sham group (gSham). Randomization will be carried out in a 1:1 ratio with permutation blocks of size unknown to the investigators. Each participant will have an equal probability of being assigned to either group. Evaluators, therapists, and participants will be blinded to the group allocations, as will the person responsible for statistical analysis.

The planned sample will seek a balanced distribution between the sexes whenever possible, recognizing the influence of the variations in the effects of transcranial stimulation due to the anatomy of the skull, cortical excitability, and neuroplasticity. The HD-tDCS device is configured to allow a preset stimulus model with specific timing and intensity codes, which cannot be identified during intervention. These codes, associated with each intervention group, will be programmed and provided by an external researcher to maintain blinding.

### 2.3. Eligibility Criteria

Participants will be included in the study if they meet the following inclusion criteria: individuals of both sexes with a clinical diagnosis of ALS according to the revised El Escorial criteria, aged between 18 and 80, Forced Vital Capacity (FVC) > 50% of the predicted value, sniff nasal inspiratory pressure (SNIP) > 40 cmH_2_O, and access to a telephone for contact with the support team.

Exclusion criteria include individuals who are unable to understand or complete any study steps; do not wish to participate or voluntarily withdraw at any time; have cardiac, chronic respiratory diseases or musculoskeletal comorbidities; are on invasive mechanical ventilation; have a tracheostomy; use a pacemaker, cerebral metal implants, or other electronic implants; have a cochlear implant; have epilepsy or a family history of epilepsy; have a history of stroke or tumors; have a high risk of severe hemodynamic variation, acute infectious, or inflammatory processes; are pregnant at the time of data collection; or do not complete the intervention protocol.

### 2.4. Interventions

Participants in the gTDCS will receive anodal high-definition direct current stimulation at an intensity of 3 mA on the diaphragmatic motor cortex through electrodes fixed in a cap. The stimulation will begin with a 30 s ramp-up to reach the intended intensity, followed by a 30 min session, and ending with a 30 s ramp-down. This will be conducted daily for 2 weeks (10 days), excluding weekends, at the same time each day in the participant’s home. The anodal current was chosen for motor cortex stimulation due to its potential for functional modulation, without directly inducing action potentials or neuronal hyperexcitability [25]. The intervention will be administered by a trained research professional with experience in neuromodulation techniques.

In the gSham, individuals will undergo all steps of the intervention and assessment protocol, but stimulation will be set to reach an intensity of 0.1 mA after the initial 30 s ramp-up, with the 3 mA current only returning in the last 30 s, resulting in no significant intervention during the 30 min.

#### HD-tDCS Protocol and Patient Familiarization

After being introduced to the HD-tDCS device, patients will receive explanations about the intervention protocol based on the application code for their assigned intervention group, along with instructions on their positioning and expected behavior during the application. Next, an adaptation and simulation protocol for HD-tDCS is conducted. The application area will be marked on a cap that the patient will wear during the intervention.

To accurately determine the stimulation site, individual structural Magnetic Resonance Imaging (MRI) data obtained during the first evaluation will be processed using the HD-Explore v 8.0.0 (Soterix Medical, New York, NY, USA) software for electric field modeling. This computational approach will guide the precise placement of electrodes to ensure targeted stimulation of the left primary motor cortex (M1) of the diaphragm [15]. The stimulation site will be identified as 1 cm anterior to the primary motor area, corresponding to the C3 position in the 10–20 EEG system, following established localization procedures.

The final electrode montage will be optimized using HD-Targets (Soterix Medical, New York, NY, USA), aligning with Montreal Neurological Institute (MNI) coordinates. The anode will be positioned over the diaphragmatic motor cortex region (3 mA), surrounded by four cathodes (0.75 mA) within a 75 mm radius from the central electrode, ensuring focalized current delivery. Once determined, the stimulation site will be marked on a cap worn by the patient throughout the intervention to maintain consistency across sessions.

For the HD-tDCS4x1 sessions, the 1 × 1 mini-CT Stimulator Model 1601 neurostimulator will be used, coupled with the 4x1-C3A HD-tDCS Multi-Channel adapter Explore (Soterix Medical, New York, NY, USA), which includes a kit with electrodes, plastic enclosures, conductive gel, elastic caps, and connector cables. To prevent electrode oxidation, all procedures will include careful cleaning, inspection, and the application of a consistent amount of conductive gel.

**Figure 2:**
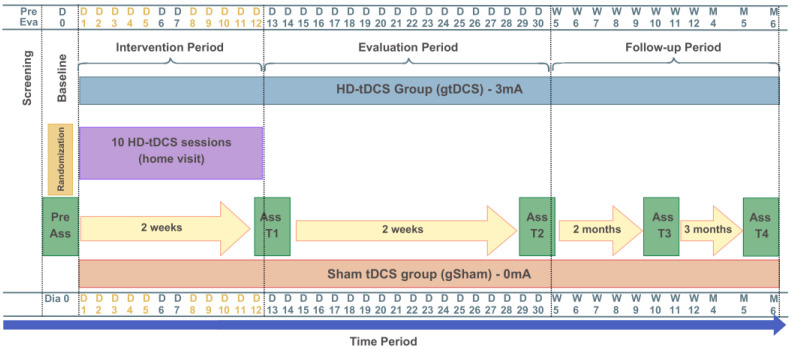
Study design: intervention and assessment protocol. The yellow letters and numbers represent the active intervention days (Days 1 to 12).

Participants will be reassessed after 10 sessions/two weeks of intervention (T1), one month after the start of the intervention (T2), three months after the intervention (T3), and six months after the end of the interventions (T4). At each session, monitoring will be conducted to assess any side effects or adverse events, or freely through phone calls if the participant finds it necessary. Adverse events will be considered as any unfavorable medical occurrence in a participant, including any abnormal sign, symptom, or disease temporarily associated with the participant’s involvement in the research. No changes will be made to any ongoing standard clinical treatment. The intervention that the gtDCS group will receive consists of 10 tDCS sessions, 5 times per week, over two consecutive weeks, with each session lasting 32 min (Figure 2).

### 2.5. Procedures and Outcomes

**Figure 3:**
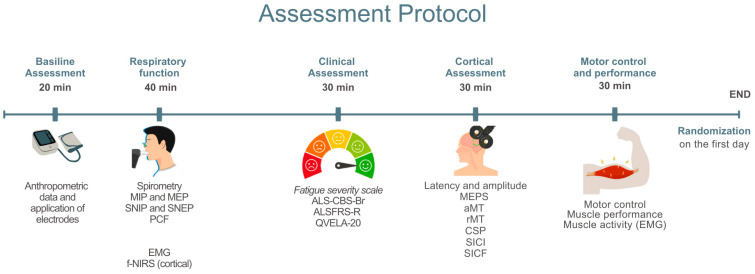
Baseline assessment. MIP: Maximum Inspiratory Pressure; MEP: Maximum Expiratory Pressure; SNIP: Sniff Nasal Inspiratory Pressure; SNEP: Sniff Nasal Expiratory Pressure; PCF: Peak Cough Flow; sEMG: Surface Electromyography; NIRS: Near-Infrared Spectroscopy; fNIRS: Functional Near-Infrared Spectroscopy; ALS-CBS-Br: Amyotrophic Lateral Sclerosis Cognitive Behavioral Screen; ALSFRS-R/BR: Revised Amyotrophic Lateral Sclerosis Functional Rating Scale—Brazilian Version; QVELA-20/Br: Short Specific Quality of Life Questionnaire for ALS Patients; MEP: Motor Evoked Potential; aMT: Active Motor Threshold; rMT: Resting Motor Threshold; CSP: Cortical Silent Period; SICI: Short-Interval Intracortical Inhibition; SICF: Short-Interval Intracortical Facilitation.

The procedures in this study will be conducted by trained professionals affiliated with the multidisciplinary team at each study center. After agreeing to participate and signing the Informed Consent Form (ICF) (Supplementary Materials S1), participants will undergo a baseline assessment (Figure 3).

A standardized evaluation form will be used for data collection on identification, characterization, and verification of inclusion and exclusion criteria. This evaluation form will include data related to anthropometric assessment (weight, height, body mass index), clinical assessment (diagnosis, type of ALS, symptoms, medications in use), use of non-invasive ventilation (NIV), and engagement in respiratory and motor physiotherapy and its frequency, in addition to monitoring of vital signs [heart rate (HR), systemic blood pressure (BP), and peripheral oxygen saturation (SpO_2_)].

Next, electrodes and sensors will be placed on the muscles to be analyzed, both for electromyography and for functional spectroscopy. Patients will undergo evaluations of pulmonary function, Peak Cough Flow (PCF), Maximum Inspiratory Pressure (MIP), Maximum Expiratory Pressure (MEP), Sniff Nasal Inspiratory Pressure (SNIP), and Sniff Nasal Expiratory Pressure (SNEP). During the execution of the respiratory muscle strength curves, cerebral perfusion [functional near-infrared spectroscopy (fNIRS)] and electromyography of the respiratory muscles will be recorded.

#### 2.5.1. Pulmonary Function

All respiratory assessments will be conducted using the PowerLab C data acquisition system (ADInstruments, Bella Vista, Australia), ensuring standardized measurements across all tests. The system will be used in conjunction with specific accessories and calibrated according to the manufacturer’s instructions prior to each session.

Pulmonary function tests will be conducted during the baseline assessment to characterize the sample and as a secondary outcome to observe the effect of the applied intervention. Measures will be taken from flow–volume curves recorded in the seated position during standard spirometry. The technical procedures and equipment standardization will follow American Thoracic Society and European Respiratory Society (ATS/ERS) recommendations. Each individual will perform the test in a seated position on a comfortable chair using a nasal clip. Examiners will provide prior guidance on correctly performing the maneuver, which involves taking a maximum inspiration close to Total Lung Capacity (TLC) followed by a rapid, sustained expiration until the examiner signals to stop the maneuver.

During spirometry, the following variables will be assessed: Forced Vital Capacity (FVC), Forced Expiratory Volume in the first second (FEV1), Forced Expiratory Flow between 25 and 75% of the FVC curve (FEF25–75%), and the FEV1/FVC ratio. The assessment will be considered complete when three acceptable curves are obtained, with at least two being reproducible. The equipment used will be a spirometry extension. Daily calibration will be performed by injecting a 3000 mL volume of air through a syringe with a 3 L calibration pump. The values obtained will be compared to relative and absolute values for the Brazilian population [37].

The assessment of inspiratory and expiratory muscle strength will be obtained through maximum respiratory pressures (MIP and MEP, respectively) using a portable mouth pressure gauge connected to the PowerLab C system transducer (ADInstruments, Bella Vista, Australia), following the European Respiratory Society guidelines. Sitting in a chair with the backrest adjusted to 90° and the right arm supported, with the nostrils occluded by a nasal clip, the subject will be instructed to exhale to residual volume and then inhale with maximal effort for at least 1.5 s. A minimum of three reproducible and acceptable measurements will be performed; if the last maneuver is the highest, a new maneuver should be performed, and variability should not exceed 10% [38].

The nasal inspiratory pressure (SNIP test) will be performed with the participant in the same position as the previous tests. The subject will be asked, at the end of a quiet exhalation (functional residual capacity), to take maximum inhalation with one nostril occluded by a plug connected to a catheter attached to a manometer, while the other nostril remains free. The subject will perform a maneuver starting from functional residual capacity, with a closed mouth, taking a maximal “sniff” through the free nostril after a slow and relaxed exhalation. The maneuver is accepted when the maximum inspiration time is less than or equal to 0.5 s. Ten SNIP maneuvers will be conducted, each separated by a 30 s rest period [39].

The nasal expiratory pressure (SNEP) will be obtained under the same conditions as above, with the subject instructed, at the end of a quiet inhalation (total lung capacity), to perform a maximum exhalation using a nasal mask connected to a catheter attached to the manometer. The subject will be directed to perform a maneuver starting from total lung capacity, with the mouth closed, performing a forced exhalation through the nasal mask, preceded by a slow, deep inhalation. Ten SNEP maneuvers will be conducted, each separated by a 30 s rest period [40].

The maximum expiratory flow will be measured during a coughing maneuver, defining the peak expiratory flow (PEF). The PowerLabC system (ADInstruments, Bella Vista, Australia) equipped with a respiratory flow head will also be used to measure this.

Before each examination, subjects will be instructed on the procedures. Evaluators will demonstrate how the maneuver should be performed. The technical criteria for acceptability and reproducibility of maximum respiratory pressure tests will follow American Thoracic Society (ATS) and European Respiratory Society (ERS) guidelines. Initially, individuals will be asked to perform a learning maneuver, and the evaluation will be considered complete when there is less than a 10% variation between the two maximum values. Predicted values will be calculated using regression equations for calculating maximum pressures based on age and gender, as proposed for the Brazilian population [41,42].

#### 2.5.2. Surface Electromyography (sEMG) of Respiratory Muscles

**Figure 4:**
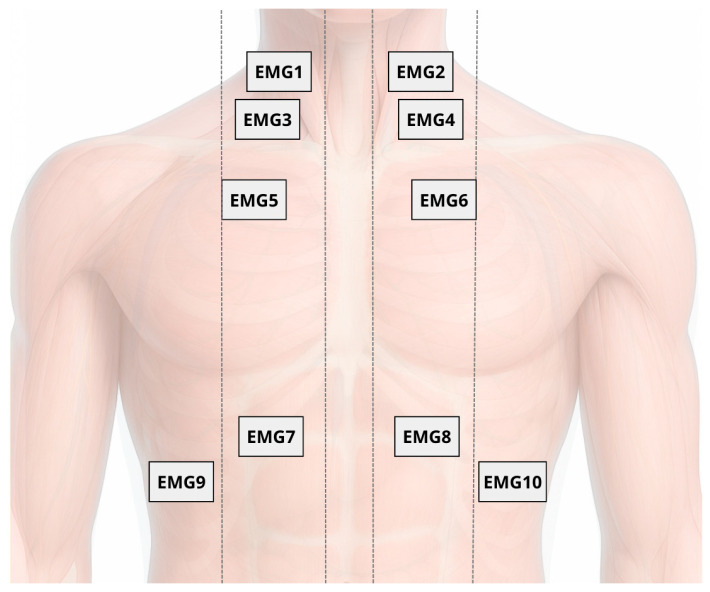
Placement of 10 surface electromyography (sEMG) electrodes on respiratory muscles: scalene (EMG1 and EMG2), sternocleidomastoid (EMG3 and EMG4), parasternal (EMG5 and EMG6), and hemidiaphragms (EMG7 to EMG10), bilaterally positioned according to SENIAM guidelines.

Surface electromyography of the respiratory muscles will be used for acquiring and processing muscle depolarization data during respiratory curves, to characterize and quantify the recruitment level for each muscle during maneuvers. This will be conducted with 10 sEMG surface electrodes (Delsys Inc., Boston, MA, USA): 2 sensors placed on the scalene (ESC) muscles (5 cm from the sternoclavicular joint and 2 cm above this point), 2 sensors on the sternocleidomastoid (SCM) muscles (in the lower third of the distance between the mastoid process and the sternoclavicular joint), 2 sensors on the parasternal (PARA) muscles (in the second intercostal space, 3 cm from the sternum), and 4 sensors on the hemidiaphragms (H-DIA) (seventh and eighth intercostal space, one electrode between the costochondral junction and the midclavicular line, and another between the midclavicular line and the anterior axillary line). All sensors will be placed bilaterally to capture myoelectric data. Skin preparation will include trimming, cleaning with 70% alcohol, gentle abrasion with gauze, and electrode placement along muscle fiber orientation following the guidelines of the Surface Electromyography for the Non-Invasive Assessment of Muscles (SENIAM) [43] (Figure 4).

The selection of the scalene, sternocleidomastoid, parasternal, and hemidiaphragm muscles aims to capture the recruitment pattern of respiratory muscles under cortical control. Although the primary target of stimulation is the diaphragmatic motor cortex, its activation is not limited to the diaphragm and may also modulate accessory muscle activity during active breathing. Therefore, the selected muscle set provides a comprehensive assessment of the respiratory motor response induced by neuromodulation.

Sensors will be affixed to the skin using double-sided tape and transpore tape over the casing to minimize motion artifacts during the test. Signals are transmitted via Bluetooth through the EMGworks Acquisition software, version 4.8 (Delsys Inc., Boston, MA, USA) for standardized data acquisition and signal analysis. The sEMG signals will be processed using EMGWorks software version 4.8 for analysis, but for the evaluation of the diaphragm sensors will be used that allow for motor unit analysis through the Neuromap software, version 1.2.1 (Delsys Inc., Boston, MA, USA), accurately reporting the location of each motor unit according to a method that decomposes, synthesizes, and compares a physiological signal synthesized from the action potentials of the motor units obtained. The algorithm inspects the signal for clear and uncontaminated shapes of the motor units and then uses these shapes to identify all the firing locations of the motor units in the sEMG signal, including where the motor units overlap, allowing for the identification of the mean amplitude of the action potential, the firing threshold, and the maximum and average firing rates of the motor units [44,45,46].

Muscle electrical activity will also be monitored at rest (before maneuvers) and during recovery (after maneuvers). The highest values obtained from active MIP and MEP maneuvers will be used to normalize the electromyographic data based on peak analysis.

#### 2.5.3. Functional Near-Infrared Spectroscopy (fNIRS)

**Figure 5:**
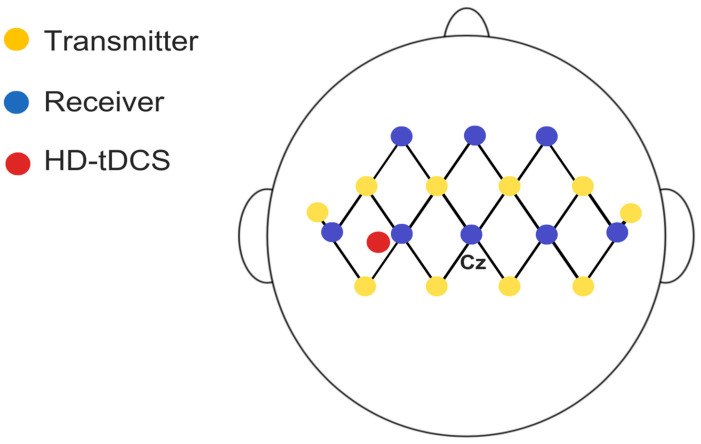
Schematic representation of fNIRS setup with 22 channels and 2 short-separation channels (10 mm), distributed across eight receivers and ten transmitters with a 3 cm distance between them in the assessed region.

Functional Near-Infrared Spectroscopy (fNIRS) is a non-invasive neuroimaging technique that uses light in the near-infrared range, applying principles of absorption and scattering based on spatially resolved spectroscopy, to monitor brain activity by detecting local changes in blood oxygenation levels [47,48,49]. Hemodynamic changes in the diaphragmatic motor cortex (M1d) will be assessed during maximal respiratory activities using the Brite MKIII portable near-infrared brain imaging system (Artinis Corporation, Elst, the Netherlands). Measurements will be taken from 27 channels configured in 10 transmitters and 8 receivers mounted in a 3 × 7 grid attached to the participant’s head with an elastic band, with the central detector placed at Fz (10–20 system), and a 30 mm distance between each emitter and detector (Figure 5). Channels will be grouped to track perfusion in the diaphragmatic motor cortex. The OxySoft version 4.0 data acquisition and analysis software will be used for synchronized data collection. Light sources emit at 750 and 830 nm, and the sampling frequency will be set at 25 Hz. Average values of Oxy-Hb, Deoxy-Hb, and total hemoglobin concentrations in each channel will be assessed during rest and respiratory curve phases.

#### 2.5.4. Cortical Excitability—Transcranial Magnetic Stimulation (TMS)

Cortical excitability will be assessed using a Neuro-MS TMS device (Neurosoft Ltd., Ivanovo, Russia). MEPs will be recorded via surface electromyography (sEMG) from the hemidiaphragms, with activity captured using a NEURO-MEP amplifier (Neurosoft Ltd., Russia) with a 5 Hz low-pass filter and a 50 mV acquisition range. Stimulation will be non-invasively and painlessly administered with a Neuro-MS device connected to an “8”-shaped coil placed over the diaphragmatic motor cortex in the left hemisphere. The vertex and interaural and nasion–inion lines will be marked on a cap based on the 10/20 system, with a 1 × 1 cm grid drawn as a TMS reference [50,51].

The left primary motor cortex “hotspot” will be identified as the scalp location eliciting MEPs ≥ 50 μV in the respiratory muscles in at least 5 of 10 stimulations at the end of expiration, and during inspiration, respectively. For all TMS pulses, the “8”-shaped coil connected to a magnetic stimulator will initially target the primary diaphragmatic motor cortex, approximately 3 cm to the left of the midline and 2–3 cm anterior to the auricular plane [15,51]. The coil will be held by the operator and angled at 45° to the midline, with the handle pointing posteriorly, generating a posterior–anterior current flow in the brain during monophasic pulse delivery [52].

Surface EMG electrodes will be positioned bilaterally on the 7th and 8th intercostal spaces to monitor diaphragmatic motor responses [53,54]. TMS will be delivered at 80% of the maximum magnetic output at end-expiration, with participants in the sitting at 45° position. Diaphragmatic EMG will be continuously monitored to guide coil orientation, which will be adjusted around the stimulation hotspot to elicit the largest Motor Evoked Potentials (MEPs) during inspiration. Stimulation will be repeated five times at 45–60 s intervals, with the three highest-quality traces selected for analysis.

**Figure 6:**
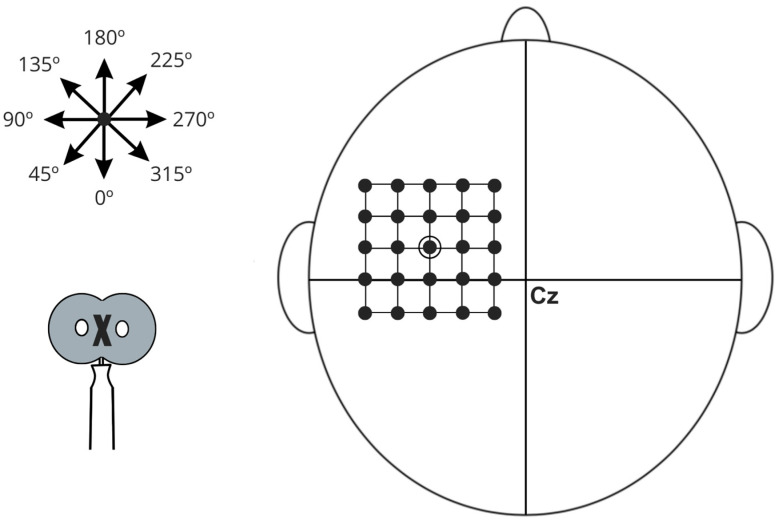
Schematic illustration of diaphragmatic motor cortex stimulation, referenced from point Cz according to the International 10–20 EEG electrode placement system. The diagram features a grid with 1 cm spacing used to identify regions of maximal muscle action potentials. A star symbol indicates various stimulation angles, while an arrow demonstrates the direction of the central current. The stimulation coil is represented at the bottom of the schematic, with an “X” denoting the aligned portion positioned over specific grid locations.

The cortical target will be identified and marked on the cap as the first grid location and explored in adjacent directions until optimal responses are obtained. To ensure accurate and consistent segmentation, the assessment will be processed using HD-Explore software (Soterix Medical, New York, NY, USA) for electric field modeling. The MRI image will be uploaded to the platform server, and the resulting individual model will be automatically integrated into the software for personalized anatomical mapping. Individual anatomical MRI scans will retrospectively confirm that stimulation was applied over the diaphragmatic motor cortex, located near the midline of the precentral gyrus, approximately 3 cm lateral to the vertex, as first described by Maskill et al. [51] and later investigated in studies on diaphragmatic corticospinal excitability in COPD and COVID-19 [15,55,56,57] (Figure 6). While absolute anatomical certainty is not achievable without combined TMS-fMRI, this protocol employs advanced neuronavigational procedures to maximize targeting accuracy.

After localization, the following will be evaluated: latency, amplitude, resting motor threshold (rMT), active motor threshold (aMT), corticospinal excitability, and cortical silent period (CSP). MEPs will be expressed as a percentage of average amplitude and analyzed for latency and area. Intracortical inhibition (SICI, LICI at 2 ms) and intracortical facilitation (SICF at 15 ms) will be assessed using 10 pulses (minimum 7) at 90% aMT [58,59,60].

### 2.6. Secondary Outcomes

#### 2.6.1. Functional Capacity

The functionality of patients with ALS will be assessed using the Amyotrophic Lateral Sclerosis Functional Rating Scale-Revised (ALSFRS-R/BR), which has been validated for the Portuguese language [61]. This scale is a specific functional assessment tool for ALS, considered clinically significant, and its score is directly related to the survival of these individuals [62]. The ALSFRS-R/BR contains 12 items, with scores ranging from 0 to 4, and its total score can vary from 0 to 48, where 48 represents normal functionality and 0 indicates severe disability [63]. The scale is divided into 4 domains, each with its respective variables: (1) bulbar function—speech, salivation, and swallowing; (2) fine motor function—handwriting, food cutting, and utensil handling (with or without gastrostomy), dressing, and hygiene; (3) gross motor function—turning in bed and adjusting bedding, walking, and climbing stairs; and (4) respiratory function—breathing, orthopnea, and respiratory failure [64].

#### 2.6.2. Motor Control and Muscle Performance

The analysis of motor control and muscle performance will follow a protocol to assess muscle activity via surface electromyography (sEMG), using the TRIGNO™ Wireless System, Delsys Inc., based on the amplitude and frequency of the muscle’s electrical activity. The TRIGNO^TM^ consists of electrodes for surface electromyography collection, as well as inertial measurement units, including an accelerometer, gyroscope, and magnetometer. Sixteen wireless sensors will be used, each measuring 37 mm × 26 mm × 15 mm in a rectangular shape, with a sampling frequency of 1926 Hz for sensors number 1–12 and 1111 Hz for sensors number 13–16. The electrodes will consist of four pure silver contact bars, measuring 5 mm × 1 mm, with an inter-electrode distance of 10 mm. They will have a passband filter of 20–450 Hz, a capture range of 40 m, and an sEMG signal resolution of 16 bits [65].

The electrodes will be positioned perpendicular to the muscle fibers and fixed with double-sided adhesive. After hair removal and cleaning the area with alcohol, the sensors will be placed on the lower trapezius, anterior deltoid, biceps brachii (medial head), triceps brachii (lateral head), brachioradialis, long radial extensor of the wrist, short abductor of the thumb, and upper trapezius muscles, bilaterally [65]. The signal will be acquired over 25 s in three phases: initial rest (R1), activity (gestures and object handling), and final rest (R2). The acquisition during gestures (G) and object handling (M) activities will occur in two stages: pre-activity rest (5 s) and the execution of the activity itself (20 s) for each task. Bimanual tasks, such as gestures and object handling, will be performed first with the right upper limb, followed by the left upper limb. The selected tasks simulate those performed in daily living activities [65].

#### 2.6.3. Fatigue and Dyspnea

Fatigue will be assessed using the Fatigue Severity Scale [66] in its Portuguese-translated version. This is a self-administered scale that aims to describe the severity and impact of fatigue on the individuals’ daily life activities over the last two weeks. It consists of nine statements, where for each item, the patient is instructed to choose a score ranging from 1 to 7, with 7 being the maximum level of agreement with the statement. The total EGF score is determined by calculating the average of all items, with a minimum score of 9 and a maximum score of 63. Scores of 28 or higher indicate significant fatigue [50,67,68,69,70,71,72]. Additionally, fatigue will be observed through muscle electrical activation captured by surface sEMG, as described above. The subjective symptom of dyspnea will be assessed using the Modified Borg Scale.

The Modified Borg Scale, unlike the original version, uses a range of 0 to 10, with each number corresponding to a textual description of the degree of respiratory discomfort (0 being minimal and 10 being maximum respiratory discomfort), in which the individual indicates their perceived level of effort [73,74]. This outcome will be assessed during pulmonary function assessment protocols, including MIP, MEP, SNIP, SNEP, and PFT curves, both active and assisted by TMS.

#### 2.6.4. Pain Assessment

The Numeric Rating Scale (NRS) and Pain Body Drawings will be used to assess pain. The NRS will be used to quantify pain intensity, with scores ranging from 0 (no pain) to 10 (maximum pain), and was chosen because it has been used in people with ALS and is an easy-to-complete tool with high reliability compared to other pain measurement scales for individuals with musculoskeletal disorders [75,76,77].

#### 2.6.5. Sleep Monitoring

To assess the frequency of sleep-related complaints, the Mini-Sleep Questionnaire (MSQ), translated and validated for Brazil, will be used. It consists of ten questions, and its score ranges from 10 to 70, where 10–24 indicates good sleep, 25–27 indicates slightly altered sleep, 28–30 indicates moderately altered sleep, and a score above 30 indicates severely altered sleep [78,79,80].

#### 2.6.6. Cognitive Assessment

The cognitive level of the patients will be assessed using the Amyotrophic Lateral Sclerosis Cognitive Behavioral Screen in its validated version for the Brazilian population—ALS-CBS-Br. This tool consists of four blocks of questions and is used for screening cognitive changes of a frontotemporal nature, with a focus on executive functions, assessing aspects such as attention, concentration, tracking and recall, and fluency. The maximum score of the test is 20 points, and scores below 10 are interpreted as a potential presence of cognitive impairment [81].

#### 2.6.7. Quality of Life

Quality of life will be assessed using the Brief Specific Quality of Life Questionnaire for ALS Patients (QVELA-20/Br), developed from the cross-cultural adaptation and validation of the Amyotrophic Lateral Sclerosis-Specific Quality of Life–Short Form (ALSSQOL-SF) for the Brazilian context. The instrument covers six main domains that influence the quality of life of patients: negative emotion, interaction with people and the environment, intimacy, religiosity, physical symptoms, and bulbar function. It is an easy-to-use tool that has been previously used to assess the quality of life of ALS patients. It consists of a 20-item Likert-type questionnaire, scored from 0 to 10 points (“Strongly disagree” to “Strongly agree”), where higher final scores indicate a better quality of life for the respective patient [82].

#### 2.6.8. Monitoring and Adverse Effects

TDCS can be presented as a therapy with a low rate of adverse or side effects. The majority of studies conducted with this therapy from 1998 to 2010 did not report any adverse effects during its application [83]. These adverse effects are described as non-serious, such as changes in sensitivity at the application site, headaches, a feeling of increased temperature, itching, and discomfort (mostly attributed to the tape that secures the electrode on the scalp). Therefore, it can be said that there is a low rate of adverse effects associated with tDCS, but this may also indicate some degree of bias in the reporting of effects related to the technique [84]. Doses that could cause brain injury are in the order of 100 mA, far above those used in humans, which range from 0.8 to 3 mA [85]. The equipment used in the study is of high quality, approved by Brazilian regulations, and properly calibrated.

The literature shows a very low rate of severe side effects that could permanently harm or worsen the participant’s health condition [84]. The treatment protocol is based on recent guidelines and recommendations for application and safety in neuromodulation and will be supervised by professionals trained in clinical tDCS application. From this perspective, even in the worst-case scenario, where the proposed neuromodulation treatment shows no discernible results, participants will still benefit from physiotherapeutic treatment over the course of two weeks. If any adverse effects arise during or after the study, participants will be referred to the appropriate hospital unit, specialized in diagnosing and treating the reported concern, under the supervision of a project physician.

During the tests, heart rate (HR), peripheral arterial oxygen saturation (SpO_2_), and blood pressure (BP) will be monitored, along with the application of the BORG scale of effort before and after. All participants, whether receiving active or placebo neurostimulation, will be questioned about their experience of effects such as itching, tingling, skin redness, drowsiness, concentration problems, headaches, fatigue, dizziness, and others [21]. Additionally, they will be asked to indicate the intensity of these sensations on a scale from 1 to 4 (where 1 is none, 2 is mild, 3 is moderate, and 4 is strong) and whether these effects are related to the stimulation, using a Likert scale from 1 (no relation) to 5 (strongly related).

### 2.7. Measurement of Outcomes

The outcome measures and tools used are listed in the following table according to the intended endpoint and in the order of the proposed objectives. In addition to these data, sociodemographic information, including gender, age, marital status, education, and employment, as well as lifestyle habits such as alcohol consumption, tobacco use, hours of sleep, and clinical information such as medication use, are included (Table 1).

## 3. Ethics and Disclosure

The project was approved by the Research Ethics Committee of the Onofre Lopes University Hospital (HUOL) and Federal University of Rio Grande do Norte (UFRN), Natal, Rio Grande do Norte, Brazil (CAAE: 68121122.1.0000.5292, on 12 December 2023), and registered at Clinical Trials with the number NCT06719947. Patients will be invited to voluntarily participate in the study, and those who, after being informed about the scheduled procedures, provide their written consent by signing the ICF will be included.

All information collected from study participants will be kept confidential and stored in the laboratory’s database during and after the trial. Data will be accessed only by researchers to ensure anonymity and respect for human dignity, complying with the ethical requirements of Resolution 466/2012 of the National Health Council and the Helsinki Declaration for research involving humans.

As with most interventions, HD-tDCS may cause adverse side effects, although they may not manifest in all individuals, such as local discomfort, itching, or redness. These acute effects are temporary, and the study will continuously monitor these during the trial. If any of these side effects persist during data collection and do not subside, the study will be immediately discontinued, and the participant will be monitored in the laboratory with emergency care, if necessary, until the symptoms disappear. If vital signs show hemodynamic instability and/or symptoms do not resolve within two hours, the participant will be evaluated by the emergency team at the hospital. Additionally, researchers will provide contact information for participants to report any adverse effects that may occur outside the data collection location, and the necessary assistance will be provided.

Not recruiting. Recruitment is scheduled to begin on 31 July 2025 and end on 31 July 2026.

## 4. Discussion

The study is in the equipment testing and improvement phase. Recruitment will begin at the centers in July 2025 and is expected to conclude in July 2026. The study will conclude once the final endpoint data have been collected from the last participant, with an expected protocol completion date of 31 December 2026. However, early termination of the study may occur following a serious adverse event that is considered linked to the intervention.

A potential limitation of this study is the relatively small sample size, given the rarity of ALS and the challenges in recruiting participants with specific clinical profiles across the centers. Despite this, a sample size of 20 individuals is estimated at each research center, totaling 60 participants. The sample size will be determined through a power analysis based on preliminary results from a pilot study and existing literature. The calculation will account for potential dropouts and instances in which reliable neurophysiological responses cannot be obtained. This approach aligns with previous studies (e.g., Benussi et al., [30]), which have used preliminary data to ensure adequate statistical power (1–β = 0.80) with a significance level of 0.05 to detect clinically meaningful effects. This could affect the statistical power of the analyses, limiting the generalizability of the results. Additionally, while the intervention (HD-tDCS) has shown promising effects in other contexts, the specific impact on respiratory outcomes in ALS requires careful consideration, as the disease’s progression can vary widely across individuals. The equipment used for data collection, including f-NIRS, sEMG, and TMS, may also introduce variability and artifacts that could influence the reliability of measurements, particularly when considering the sensitivity of these technologies to movement or changes in physiological conditions.

Although techniques such as neuronavigation and real-time functional neuroimaging allow for more precise stimulation, their absence does not preclude the clinical application of neuromodulation. Previous studies indicate that stimulation based on standardized anatomical coordinates—even without neuronavigation—can produce physiologically relevant effects, especially when the target is the primary motor cortex, whose location is relatively well defined. However, anatomical variability among individuals, both in cortical positioning and in the functional representation of the diaphragm, may influence individual responses to the intervention. This variability underscores the importance of complementary functional monitoring, such as the use of functional neuroimaging techniques (fNIRS, EEG) or physiological measurements (respiratory pressures, electromyography), to validate the response to the protocol. Moreover, if no statistically significant differences are observed between the gSham and gTDCS groups, the findings will still yield important insights regarding the feasibility, safety, and methodological challenges of implementing HD-tDCS in individuals with ALS. These results may guide future adjustments to stimulation parameters, participant stratification strategies, or the incorporation of additional biomarkers.

Therefore, the selection of a cortical target—particularly the diaphragmatic primary motor cortex—is supported by anatomical, functional, and clinical evidence. The strategy aims to modulate cortical respiratory drive, enhance sensorimotor integration, and provide compensatory support in the face of the progressive failure of lower motor neurons—a central mechanism in the pathophysiology of ALS.

In terms of dissemination, the results of the study will be shared through peer-reviewed publications, as well as presentations at national and international conferences, particularly those focused on ALS, neurostimulation, and respiratory therapies. Additionally, we plan to make the data publicly available in open-access repositories to ensure transparency and foster further research in this field.

## 5. Limitations

Among the main limitations of this study is the lack of well-established guidelines for applying TMS in assessment protocols targeting respiratory function. The scarcity of specific evidence to guide the standardization of stimulation parameters and coil positioning necessitates methodological adjustments that will first be tested in a pilot study, aiming to minimize systematic errors and improve data reproducibility.

Additionally, individual variability in both the anatomy of the diaphragm and its electromyographic activation (sEMG) represents a significant challenge, potentially influencing the response to the intervention and the consistency of measurements. This anatomical heterogeneity may hinder the accurate localization of the diaphragmatic motor cortex, particularly in the absence of neuronavigation technologies, which could provide greater precision in identifying the cortical target. Although the use of standardized anatomical coordinates is a feasible and evidence-supported alternative, this approach may not adequately account for interindividual differences in the functional representation of the diaphragm.

These factors highlight the importance of complementary monitoring strategies, such as the use of physiological markers and functional neuroimaging techniques (fNIRS, EEG), to appropriately validate and interpret the effects of the intervention.

## Figures and Tables

**Table 1:** Results, measurements, and analysis instruments according to objectives.

Outcome	Measures	Instrument
Cortical excitability	Latency and amplitude MEP—Motor Evoked Potential LMr—Rest Motor Threshold LMa—Active Motor Threshold CSP—Cortical Silent Period CMCT—Central Motor Conduction Time CIF—Intracortical Facilitation LICI—Long Interval Intracortical Inhibition SICI—Short Interval Intracortical Inhibition	TMS
Cerebral perfusion	HbDiff—Hemoglobin concentration difference at wavelength tHb—Total hemoglobin concentration at wavelength HHb—Deoxygenated hemoglobin concentration at wavelength O2Hb—Oxygenated hemoglobin concentration at wavelength TSI %—Tissue Saturation Index	f-NIRS
Respiratory muscular electrical activity	RMS (Root Mean Square)—Signal amplitude in microvolts Standardization of the signal in % RMS FM—Median Frequency Motor unit activation (recruitment) Neuronal firing Fatigue development	sEMG
Nasal pressures	SNIP—Maximum Inspiratory Nasal Pressure SNEP—Maximum Expiratory Nasal Pressure	PowerLab C
Cough peak flow	Maximum expiratory flow measured during a cough maneuver	PowerLab C
Functionality	Bulbar function—speech, salivation, swallowing; fine motor function—handwriting, food cutting, utensil handling; gross motor function—bed mobility, walking, stair climbing; respiratory function—breathing, orthopnea, respiratory failure	ALSFRS-R
Motor control and performance	Protocol for evaluating muscle activity through %RMS during rest, gestures, and object handling	sEMG
Fatigue	Nine statements, each item scored from 1 to 7, where 7 indicates maximum agreement with the statement	Fatigue Severity Scale
Fatigue/dyspnea	Scale from 0–10	Borg scale
Pain	Presence, intensity, and location of pain, with scores from 0 (no pain) to 10 (maximum pain) and body diagrams	Numerical Rating Scale (NRS) and Pain Body Drawings
Sleep	Frequency of sleep complaints via ten questions with scores ranging from 10 to 70, where 10–24 indicates good sleep, 25–27 mildly altered sleep, 28–30 moderately altered sleep, and over 30 indicates highly altered sleep	MSQ
Quality of life	20 items related to physical, emotional, social, spiritual, and financial aspects	QVELA-20/Br
Cognitive assessment	Attention, concentration, tracking and recall, fluency	ALS-CBS-Br
Adverse effects	Presence and severity	Subjective
Lung function	FVC—Forced Vital Capacity FEV1—Forced Expiratory Volume in the first second FEV1/FVC—FEV1/FVC ratio FEF25–75%—Forced Expiratory Flow at 25–75% range	PowerLab C
Vital signs	HR—heart rate RR—respiratory rate SaO_2_—arterial oxygen saturation BP—blood pressure	Digital oximeter and sphygmomanometer

Notes: TMS (Transcranial Magnetic Stimulation), sEMG (Surface Electromyography), fNIRS (Functional Near-Infrared Spectroscopy), ALSFRS-R (Amyotrophic Lateral Sclerosis Functional Rating Scale-Revised), MSQ (Mini-Sleep Questionnaire), QVELA-20/Br (Quality of Life Brief Questionnaire for ALS Patients), and ALS-CBS-Br (Amyotrophic Lateral Sclerosis Cognitive Behavioral Screen).

## Data Availability

No new data were created or analyzed in this study. Data sharing is not applicable to this article.

## Supplementary Materials

The following supporting information can be downloaded at: https://www.mdpi.com/article/10.3390/jcm14196701/s1, S1—Informed Consent Form (ICF); S2—Checklist. SPIRIT 2013 checklist: Recommended items to address in a clinical trial protocol and related documents; S3—Full Study Protocol Approved by the Ethics Committee (English Translation).

## Abbreviations

The following abbreviations are used in this manuscript:

ALS	Amyotrophic Lateral Sclerosis
ALS-CBS-Br	Amyotrophic Lateral Sclerosis Cognitive Behavioral Screen—Brazilian version
ALSFRS-R	Revised Amyotrophic Lateral Sclerosis Functional Rating Scale
BP	Blood Pressure
CONSORT	Consolidated Standards of Reporting Trials
CSP	Cortical Silent Period
EEG	Electroencephalogram
fNIRS	Functional Near-Infrared Spectroscopy
FVC	Forced Vital Capacity
gSham	Sham Group
gTDCS	tDCS Group
HD-tDCS	High-Definition Transcranial Direct Current Stimulation
HR	Heart Rate
ICF	Informed Consent Form
M1	Primary Motor Cortex
MEP	Motor Evoked Potential
MEPs	Maximum Expiratory Pressure (context-dependent)
MIP	Maximum Inspiratory Pressure
MNI	Montreal Neurological Institute
MRI	Magnetic Resonance Imaging
NIV	Non-Invasive Ventilation
PCF	Peak Cough Flow
QVELA-Br	Short Specific Quality of Life Questionnaire for ALS Patients—Brazilian version
rMT	Resting Motor Threshold
SICI	Short-Interval Intracortical Inhibition
SICF	Short-Interval Intracortical Facilitation
SNIP	Sniff Nasal Inspiratory Pressure
SNEP	Sniff Nasal Expiratory Pressure
sEMG	Surface Electromyography
SpO_2_	Peripheral Oxygen Saturation
SPIRIT	Standard Protocol Items: Recommendations for Interventional Trials
tDCS	Transcranial Direct Current Stimulation
TMS	Transcranial Magnetic Stimulation
