# Supplementary material for: High-Definition Transcranial Direct Current Stimulation (HD-tDCS) Therapy in Amyotrophic Lateral Sclerosis: Study Protocol for a Multicenter Randomized Controlled Clinical Trial
Source: J Clin Med. 2025 Sep 23;14(19):6701. doi: 10.3390/jcm14196701 (PMC12525124; doi:10.3390/jcm14196701)
Supplement: Supplementary file 1 [file jcm-14-06701-s001.zip › jcm-3685550-S1.pdf]

INFORMED CONSENT FORM (ICF)

Clarifications

This is an invitation for you to take part in the research: **HIGH-DEFINITION TRANSCRANIAL DIRECT CURRENT STIMULATION (HD-tDCS) IN AMYOTROPHIC LATERAL SCLEROSIS: A RANDOMIZED MULTICENTER CONTROLLED CLINICAL TRIAL**, which has Edna Karla Ferreira Laurentino as the researcher in charge.

This research aims to verify the effects of Transcranial Direct Current Stimulation (HD-tDCS), which is a low-intensity electrical stimulus on the diaphragmatic motor cortex, in two different forms of current, anodic and cathodic, in order to evaluate the function of breathing in relation to strength and resistance, as well as observing the response of the respiratory muscles in healthy individuals.

The reason we are doing this research is the lack of studies showing the effects of these currents on respiratory functions from stimulation of the region of the brain that corresponds to this area.

If you decide to take part, you will receive HD-tDCS stimulation for an average of 30 minutes over 10 days, including assessments before, during and after the collection, which lasts an average of 3.5 hours. Electrodes will be placed at specific points on your head by properly trained professionals in order to carry out neurostimulation with a view to improving respiratory performance. Each session will take place on consecutive days at your home for two weeks, except at weekends. In addition, before and after these ten days, you will undergo a physiotherapeutic assessment with non-invasive tests to observe lung function, respiratory muscle strength and breathing patterns, and this assessment will be repeated after one month, three months and six months. This study will last an average of three and a half hours each day. The evaluations will be carried out using tests of maximum inspiration pressures, measures of lung capacity obtained by spirometry and cardiovascular function data.

During the research, you may experience discomfort and possible risks. The risk you run is similar to that felt during a physical examination. In some cases, the stimulation used may cause local itching or redness, but this acute effect has a short duration and you will therefore be at rest and monitored throughout this period. You may also experience momentary respiratory discomfort (tiredness) during respiratory assessment, due to the use of accessory muscles for breathing. These risks can be minimized and possible discomfort re-established through rest, physiotherapeutic manoeuvres and/or the use of oxygen therapy support, with our research team providing all the assistance necessary for your recovery within the hospital.

As a benefit of the research, you will receive physiotherapeutic assistance for respiratory and motor care, as well as a cardiorespiratory assessment with sophisticated equipment and qualified professionals.

In the event of complications or damage to your health related to the research, it is up to the researcher responsible to guarantee your right to full and free care, which will be provided through physiotherapeutic assistance by the researchers responsible.

Throughout the research period you can ask questions by calling Edna Karla Ferreira Laurentino, e-mail: ednakarlaferreira@gmail.com, cell: (84) 99471-5172.

You have the right to refuse to take part or to withdraw your consent at any stage of the research, without any harm to you.

If you have any questions about the ethics of this research, you should call the Research Ethics Committee - an institution that assesses the ethics of research before it begins and provides protection for participants - at the Onofre Lopes University Hospital of the Federal University of Rio Grande do Norte, on (84) 3342-5003, e-mail cep_huol@yahoo.com.br. You can also go in person to the CEP headquarters, from Monday to Friday, from 7:30 a.m. to 12:30 p.m. and from 1:30 p.m. to 3:00 p.m., at the Onofre Lopes University Hospital, address Av. Nilo Peçanha, 620 - Petrópolis – João Machado Complex – 1st Floor – Administrative Building – ZIP Code: 59012-300 – Natal/RN.

This document has been printed in two copies. One will remain with you and the other with the researcher responsible: Edna Karla Ferreira Laurentino.

Free and Informed Consent

After having been informed about the objectives, importance and the way in which the data will be collected in this research, as well as knowing the risks, discomforts and benefits that it will bring to me and being aware of all my rights, I agree to participate in the research HIGH-DEFINITION TRANSCRANIAL DIRECT CURRENT STIMULATION (HD-tDCS) IN AMYOTROPHIC LATERAL SCLEROSIS: A RANDOMIZED MULTICENTER CONTROLLED CLINICAL TRIAL and I authorize the disclosure of the information I have provided in congresses and/or scientific publications as long as no data can identify me.

____

Signature of research participant

Participant's fingerprint

Declaration by the researcher responsible

As the researcher responsible for the study HIGH-DEFINITION TRANSCRANIAL DIRECT CURRENT STIMULATION (HD-tDCS) IN AMYOTROPHIC LATERAL SCLEROSIS: A

RANDOMIZED MULTICENTER CONTROLLED CLINICAL TRIAL, I declare that I take full responsibility for faithfully complying with the methodological procedures and rights that have been clarified and assured to the participant of this study, as well as maintaining secrecy and confidentiality regarding their identity.

I also declare that I am aware that if I fail to comply with this commitment I will be in breach of the rules and guidelines proposed by Resolution 466/12 of the National Health Council (CNS), which regulates research involving human beings.

Natal/RN, ___/___/___

Signature of Responsible Researcher

____

Edna Karla Ferreira Laurentino CPF: 117.292.974-20
