# Supplementary material for: High-Definition Transcranial Direct Current Stimulation (HD-tDCS) Therapy in Amyotrophic Lateral Sclerosis: Study Protocol for a Multicenter Randomized Controlled Clinical Trial
Source: J Clin Med. 2025 Sep 23;14(19):6701. doi: 10.3390/jcm14196701 (PMC12525124; doi:10.3390/jcm14196701)
Supplement: Supplementary file 1 [file jcm-14-06701-s001.zip › jcm-3685550-S3.pdf]

S3. Schedule of enrolment, interventions, and assessments (SPIRIT).

	STUDY PERIOD							
	**Enrolment**	**Allocation**	**Post-allocation**					**Follow-up**
**TIMEPOINT****	***-w1***	**0**	***w1***	***w2***	***w3***	***m1***	***m3***	***m6***
**ENROLMENT:**								
**Eligibility screen**	X							
**Informed consent**	X							
**Allocation**		X						
**INTERVENTIONS:**								
**Active HD-tDCS**			X	X				
**Sham HD-tDCS**			X	X				
**ASSESSMENTS:**								
Baseline: *Height, Weight, BMI, Vital Signs (Temperature, BP, RR, HR, SpO_2), FVC, FEV_1, FEF_{25-75}%, FEV_1/FVC Ratio.*		X						
Primary Outcomes: *Cortical Excitability (assessed by TMS), Cerebral Perfusion (assessed by f-NIRS), Respiratory Muscular Electrical Activity (assessed by sEMG), Tissue Perfusion of Respiratory Muscles (assessed by NIRS).*		X			X	X	X	X
Secundary Outcomes: *MIP, MEP, SNIP, SNEP, PCF, Functionality (ALSFRS-R), Motor Control (sEMG), Fatigue (FSS), Pain (NRS), Sleep (MSQ), QoL (QVELA-20/Br), Cognitive (ALS-*		X			X	X	X	X

CBS-Br).								
Monitoring: *Borg RPE Scale, HR, RR, BP, SaO2, Adverse Effects.*		X	X	X	X	X	X	X

Notes: BMI (Body Mass Index), BP (Blood Pressure), RR (Respiratory Rate), HR (Heart Rate), SpO_2 (Oxygen Saturation), FVC (Forced Vital Capacity), FEV_1 (Forced Expiratory Volume in 1 Second), FEF_{25-75}% (Forced Expiratory Flow 25-75%), FEV_1/FVC Ratio, TMS (Transcranial Magnetic Stimulation), sEMG (Surface Electromyography), fNIRS (Functional Near-Infrared Spectroscopy), MIP (Maximum Inspiratory Pressure), MEP (Maximum Expiratory Pressure), SNIP (Nasal Inspiratory Pressure), SNEP (Sniff Nasal Expiratory Pressure), PCF (Peak Cough Flow), ALSFRS-R (Amyotrophic Lateral Sclerosis Functional Rating Scale-Revised), NRS (Numerical Rating Scale), MSQ (Mini-Sleep Questionnaire), QVELA-20/Br (Quality of Life Brief Questionnaire for ALS Patients), ALS-CBS-Br (Amyotrophic Lateral Sclerosis Cognitive Behavioral Screen).
